# “The Message Is You Don’t Exist”: Exploring Lived Experiences of Rural Lesbian, Gay, Bisexual, Transgender, Queer/Questioning (LGBTQ) People Utilizing Health care Services

**DOI:** 10.1177/23779608211051174

**Published:** 2021-10-16

**Authors:** Nadine R. Henriquez, Nora Ahmad

**Affiliations:** 1 Department of Nursing, Faculty of Health Studies, Brandon University, Brandon, Manitoba, Canada

**Keywords:** qualitative research < research, nursing education, LGBTQ, rural minorities, access to care

## Abstract

**Background:**

Lesbian, gay, bisexual, transgender, queer/questioning (LGBTQ) people experience significant health inequities with well-documented negative health impacts due to their status as a sexual and gender minority population. Insensitive or discriminatory attitudes toward LGBTQ people within the health care system have negatively impacted access to health services and the overall physical and mental health and well-being of this at risk population. Few studies of LGBTQ populations in rural areas have been conducted, with even fewer in the Canadian context. Rural areas often create greater visibility for LGBTQ persons, contain fewer supports and alternatives in the face of discrimination, and are often are less accepting of LGBTQ populations due to increased stigma and social isolation.

**Objective:**

The purpose of this study is to examine the lived experiences of LGBTQ people utilizing health care services in rural Manitoba.

**Method:**

12 individuals who self-identified as LGBTQ who had accessed health care services in Manitoba were recruited. Using qualitative methodology, interviews were recorded and analyzed for themes.

**Results:**

Analysis revealed themes including stigma and discrimination, judgments and assumptions, gender identities, lack of knowledge, limited access/systemic barriers, rural considerations, and recommendations for changes to address the gaps in health care services and barriers to access.

**Conclusions:**

This study of the LGBTQ community provides an expression of their opinions and experiences, but also provides a voice to this underserved population. The findings of this study provide a better understanding of the unique health needs and experiences of LGBTQ people in rural Manitoba, creating opportunities for meaningful change in health care delivery

## Introduction

Lesbian, gay, bisexual, transgender, intersex, two-spirit, and queer/questioning (LGBTQ) communities experience numerous health inequities and face many barriers when it comes to accessing health care services in Canada (House of Commons Canada [HCC], 2019). These health inequities can be attributed to the stigmatization of gender and sexual minorities and the discrimination they can face, as well as the heteronormative and cisnormative nature of society in Canada, leaving LGBTQ people feeling shame regarding their sexual orientation or gender identity (Girard in HCC, 2019). Previous negative experiences with health care services, cultural, religious, and personal beliefs can all affect a patient's comfort in accessing health care and disclosing concerns related to their sexual orientation or gender identity (National Academies of Science, Engineering, & Medicine [NASEM], 2020). Fears of victimization and stigmatization, or the feeling that providers are not competent to address their concerns, create additional barriers to accessing health care (HCC, 2019; NASEM, 2020).

Due to discrimination, harassment, and barriers to equitable health services, LGBTQ communities continue to experience higher rates of mental health concerns, including depression, anxiety, and higher rates of unhealthy coping behaviors (HCC, 2019; NASEM, 2020; The National LGBT Health Education Centre, 2016). Research shows that sexual minorities (eg, LGBTQ) experience higher levels of discrimination, stigma, and stress and are at higher risk of some poor health outcomes and health behaviors compared to their heterosexual counterparts (Jackson et al., 2016). In addition to the health disparities related to sexual orientation and gender identity, many LGBTQ people experience further health differences due to the intersection of other factors regarding their identity, including factors such as age, ethnic origin, income, and access to health care (HCC, 2019; NASEM, 2020). Lack of provider knowledge and cultural competence regarding LGBTQ health care needs and insufficient research about the health of LGBTQ populations are also major issues impacting both quality of and access to appropriate care (NASEM, 2020). Sexual and gender minorities have frequently reported needing to educate health professionals on their health needs. Moreover, health care workers have refused to treat people belonging to sexual or gender minorities because they do not feel that they are sufficiently trained, which can lengthen the time it takes to access care (Daley in HCC, 2019). Additionally, recent research has shown that many practicing health care professionals and health care trainees are often lacking knowledge, comfort, or cultural competence in addressing a variety of health issues facing LGBTQ populations (Andermann, 2016; Colpitts & Gahagan, 2016). Transgender people in particular represent one of the most marginalized groups who experience the greatest stigma and discrimination when accessing health and social services (HCC, 2019). Experiences and fears of mistreatment, confidentiality breaches, reluctance to disclose status, and needing to teach health care providers about trans care have been reported (Bell & Purkey, 2019).

## Review of the Literature

Lee and Kanji’s (2017) literature review exploring the health care experiences and barriers to care of LGBTQ individuals in North America reported that the LGBTQ community has unique health concerns and is at higher risk for mental health conditions, substance use, and suicide. These health disparities are related to discrimination, ignorance, and assumptions about gender, sex, and sexuality, which led to delays or discontinued care, nondisclosure of sexuality or gender identity, increased negative health behaviors, and internalized stigma (Lee & Kanji, 2017). Another 2019 study of trans individual's physician experiences reported a lack of knowledge regarding trans identities and health care needs. Participants also reported that despite being provided resources, some physicians remained unwilling to provide health care services. As a result, participants not only had to seek out physicians elsewhere, and further contributed to a lack of trust in physicians and the health care system (Bell & Purkey, 2019). A nationwide survey of Canadian trans youth also found that between 33%–47% reported having failed to receive necessary health care in the past year, with 61% reporting this was due to fears of how the provider would respond to them (Veale et al., 2015).

Given the health disparities and unique challenges that LGBTQ persons experience, it is important to understand how these experiences impact overall health and well-being, and how these issues can be addressed through the removal of barriers and improved education. One major barrier is the lack of standardized education addressing the health of LGBTQ patients. Medical education in Canada related to LGBTQ health remains sparse and inconsistent, despite acknowledgment of the need to prepare trainees for compassionate and comprehensive care to this population (HCC, 2019; Schreiber et al., 2021). Recent studies examining LGBTQ theoretical content in baccalaureate nursing programs in Canada also reported that LGBTQ content was very limited and lacked standardization across institutions, resulting in inconsistent and inadequate curricula (Shortall, 2019). This lack of standardization has resulted in knowledge deficits regarding the intersection of LGBTQ identities, and how systemic barriers affect health and health care access (Schreiber et al., 2021; The National LGBT Health Education Centre, 2016).

A number of gaps in knowledge have been identified in the literature regarding LGBTQ experiences within health care settings, with even less data reported regarding the health care experiences and barriers to access that rural LGBTQ people face, providing evidence for the need for research using a rural lens (MAP, 2019; Whitehead et al., 2016). Although many of the challenges LGBTQ people face are the same for all persons living rurally, including shrinking populations, few health care providers, and reduced services and employment, they also face unique challenges. The Movement Advancement Project (2019) report describing rural life for LGBTQ people in the United States, described how the social and political environment in rural areas are often less supportive of LGBTQ people, resulting in heightened vulnerability to discrimination. Additionally, geographic distances and isolation serve to amplify these issues, making self-advocacy and the ability to effect local change even more difficult (MAP, 2019).

The purpose of this study was to examine the lived experiences of LGBTQ people accessing health care services in rural Manitoba. By involving the LGBTQ community, this study facilitates an improved understanding of the unique health needs and experiences of LGBTQ persons rurally and will facilitate the development of educational programs that promote diversity, health equity, cultural competency, and inclusive, ethically responsible services.

## Method

### Design

Phenomenological-hermeneutic perspectives guided the research design and method of this study, to help understand and attach meaning to one's lived experience. This approach was critical to gain an in-depth understanding of both the nature and impact of individual experiences. In the hermeneutic phenomenological analysis, a thematic analysis approach was used as an interpretive strategy to identify themes in the interview transcripts (Van Manen, 2014). Braun and Clarke (2013) thematic analysis method was utilized, involving seven steps: transcription, reading and familiarization, coding, searching for themes, reviewing themes, defining and naming themes, and finalizing the analysis.

### Research Questions

In order to gain a better understanding of the unique health care experiences and needs of LGBTQ people in rural Manitoba, this study sought:

1. to examine health care utilization experiences of LGBTQ persons in rural Manitoba,
2. to explore the impact of these health care experiences on the health and well-being of LGBTQ persons,
3. identify barriers to access and gaps in health care services for LGBTQ persons in rural Manitoba and,
4. to identify criteria that promote positive/negative health care interactions.

### Sample and Procedure

Individuals ages 16 + , who self-identified as being gay, lesbian, bisexual, transgender, or queer/questioning, or any other nonheterosexual/nonbinary gender identity who had accessed health care services in Manitoba were sought for recruitment. In total, twelve participants were interviewed. All interviews were recorded to ensure inter-rater reliability, and were analyzed using thematic data analysis techniques and procedures. The participants with informed consent were contacted by the researchers and were assigned a secure ID code. Interviews were conducted in-person and by telephone, at the participants’ designated place and time. Only the participant and researcher were in the room, for the duration of the interviews, which lasted between one to two hours. Data saturation was obtained with a total of 12 participants. This study used the “new information threshold” method to assess saturation levels, meaning the lower the new information threshold, the less likely an important number of themes may remain undiscovered in later interviews if data collection stops when the threshold is reached (Guest et al., 2020). Guiding interview questions and probes were used to guide the sessions (see Table 1), and demographic data were collected (see Table 2).

**Table 1.** Interview Guiding Questions.

**Please tell me about your experience and the factors that encouraged or discouraged you to access the health services.** **What are your thoughts and experiences regarding your ability to disclose your sexual identity, as a health care consumer/user? What concerns or fears regarding the reaction of health care providers toward your LGBTQ status?** **What was your reaction toward** the **presence/absence of LGBTQ equality signs in a health care setting?** **How did you feel about the word choices on health intake forms you have encountered?** **How did you feel about the cultural competency levels (level of knowledge) of health care providers regarding the LGBTQ community?** **Please share your thoughts about the barriers/discriminatory actions and attitudes of health care providers toward LGBTQ that you encountered.** **What suggestions do you have for changes or improvements you would like to see in the health care setting in terms of LGBTQ care?** **Please tell me about your experience and the factors that encouraged or discouraged you to access the health services.** **What are your thoughts and experiences regarding your ability to disclose your sexual identity, as a health care consumer/user? What concerns or fears regarding the reaction of health care providers toward your LGBTQ status?** **What was your reaction toward** the **presence/absence of LGBTQ equality signs in a health care setting?** **How did you feel about the word choices on health intake forms you have encountered?** **How did you feel about the cultural competency levels (level of knowledge) of health care providers regarding the LGBTQ community?** **Please share your thoughts about the barriers/discriminatory actions and attitudes of health care providers toward LGBTQ that you encountered.** **What suggestions do you have for changes or improvements you would like to see in the health care setting in terms of LGBTQ care?**

**Table 2.** Demographic Characteristics.

Age	Sexuality	Gender	Education	Relationship	Employment
28	Lesbian	Trans woman	Gr 12, some college	Single	Unemployed
49	Gynasexual	Nonbinary	Post baccalaureate	Married	Employed
27	Lesbian	Female	College	Married	Employed
31	Queer	Trans/nonbinary	College diploma	Common law/open	Employed
32	Gay	Trans male	College diploma	In relationship	Employed
25	Bisexual	Trans/nonbinary	High school	Open partnership	Unemployed
34	Gay/queer	Male	Bachelors	Dating	Employed
38	Lesbian	Woman/genderqueer	Masters	Monogamous partnership	Employed
29	Pansexual	Queer woman	Bachelors x 2	In relationship	Employed
36	Lesbian	Transwoman	High school	Single	Unemployed
35	Gay	Male/nonbinary	High school	Single	Unemployed
33	Queer	Nonbinary	High school	Single	Employed

### Inclusion Criteria

Broad inclusion criteria were used in this study because the LGBTQ population is known as a “hard to reach population” and according to Bishop et al. (2016), recruitment of special populations in rural communities is challenging given geographic distances, transportation barriers, and the low number of eligible participants. Research suggests that study participation rates for special populations have fallen to levels that could endanger the successful performance of some types of research (Winter et al., 2018). Purposive and snowball sampling methods were used to gather data. Partnership with the local Sexuality Education Resource Center Brandon, and the Rainbow Resource Center Manitoba and their social networks (eg, friends/acquaintances) were established to recruit through their social media platforms and offices.

### Ethical Considerations

The University's Ethics Review Committee granted ethical approval prior to data collection. The study involved few sensitive questions; a referral to an appropriate counseling service was available upon request, if participants experienced any negative consequences, such as severe emotional distress.

### Data Analysis

The Principal Investigator conducted interviews and kept notes. Individual interviews were digitally recorded and transcribed verbatim, with personal identifying information removed. Using a thematic analysis approach, both researchers conducted a line-by-line review of the transcripts and developed a coding framework based on issues that arose in the text. The transcripts were coded, themes identified, reviewed, and refined. In the final step, the major themes were expanded and subthemes identified within each major theme. The researchers reviewed the transcripts, discussed, and clarified the initial coding structure to ensure the representativeness of emerging categories. Multiple transcript reviews were conducted to enhance findings validity. Trustworthiness and credibility were achieved through prolonged engagement and clarification with participants.

## Results

A total sample of 12 participants ranging in age from 25–49 years with a variety of gender identities and sexual preferences were interviewed and demographic data were collected (see Table 2).

Data analysis resulted in the emergence of overarching themes and the experiences of participants when accessing health services. Participants shared experiences and feelings leading to six overarching themes related to experiences, including stigma and discrimination, judgments and assumptions, gender identities, lack of knowledge, limited access/systemic barriers, and rural health considerations. Participants also provided recommendations for changes in health care education and delivery.

### Stigma and Discrimination

The overarching theme of experiences of stigma and discrimination manifested in various ways, on multiple occasions for all participants. Participants reported personal experiences of perceived discrimination, as well as narratives reported by other LGBTQ persons in their community.

## Fear of Disclosure: “What Kind of Reception Am I Going to Get?”

Participants described fears in deciding whether to disclose their sexual orientation or gender identity to health care providers, based on personal past experiences disclosing. Given the frequency of LGBTQ individuals’ experiences of negative attitudes in these settings, in both overt and covert forms of discrimination, one participant described this decision as feeling like a “risk”:

It's like a risk every time you know, every time you go to the doctor it feels like a risk, whether it's a different doctor or a nurse that you don’t know or whatever, right?

(Participant #0819, pansexual queer woman, 29)

Participants also described how emotionally draining mistreatment feels: “….that's an exhausting thing to deal with - discrimination”. Others described feeling concerned for personal and emotional safety:

Just some like safety concerns about like, oh, I don’t know if we’re going to get lectured about this or do we have to explain, or more of the personal feeling of safety. You never know what someone's gonna say- What's the attitudes to folks in rural areas might hold, but we’re just anxious in rural areas but I guess just some folks meddle but… More personal feelings of safety.

(Participant #0819, pansexual queer woman, 29)

I’ve just also had so many queer and transphobic experiences in medical care that I sort of pick and choose my- or have to historically pick and choose my disclosure- based on necessity and- and emotional boundaries.

(Participant #0719, queer nonbinary trans, 31)

## Judgments and Assumptions: “Those Curious Questions”

All participants described a variety of experiences in health care settings where health care providers subjected them to judgments and assumptions. These experiences included health care providers assuming relationships were heterosexual, making assumptions on gender-based on physical appearances, and passing judgments/making derogatory remarks regarding sexual practices, lifestyle, and mental health status:

I think it's when the doctors are start telling you what you’re doing is wrong rather than listening to you. I know friends who have stories that the doctor telling them that they’re evil or they’re sinning.

(Participant #1719, gay male, 34)

The assumptions often involved heteronormative biases, or involved discriminatory attitudes. The failure of the health care provider to ask appropriate questions, or asking questions in a way that prevented disclosure or made disclosure less comfortable were reported:

I mean, even if an intake person gets it right, it's not communicated to the next person, it's not put in an obvious place on my file so I will be misgendered by doctors or nurses or you know, through a shift change or I’ll overhear (them).

(Participant #0719, queer nonbinary trans, 31)

On the opposite spectrum was the noted frequency of exhausting, intrusive questions based on curiosity, not a necessity. One participant described questions regarding sexual reassignment surgery during an assessment for a chest infection at a walk-in clinic, where such inquiries would have had no potential medical utility. Another participant described the emotional toll of such intrusive questioning:

(Physician) might be able to figure out on their own (that I’m gay) either way, but I just don’t bother- ‘cause it's not worth my energy in case they are not a safe person. It's just like if it's homophobia, that's an exhausting thing to deal with discrimination.

(Participant #1719 gay/queer male, 34)

Just tiring, frustrating, and it can last for a few days, the effects of it- if it's a bunch of questions, even if they’re positive and encouraging, that's like good, but also still just, you know, when you need to answer that one question throughout your life, you’re tired of it. Just more curious questions, yeah. Those curious questions.

(Participant #1719 gay/queer male, 34)

### Gender Identities and the Pathologization of Identity: “It Hurts Your Credibility”

All the participants who identified as transgender described the process of receiving support for transition as being “pathologized” and “medicalized.” In Canada, individuals require diagnosis under the criteria of the American Psychological Association (2013) Diagnostics and Statistical Manual (5th ed; DSM-5) with gender dysphoria to qualify for support for physical transitioning. The DSM-5 pathologized homosexuality until recent decades and continues to portray gender dysphoria as a mental health issue (American Psychological Association, 2013). Participants described these processes as troubling and “archaic” with forms listing gender stereotypes and asking the person which gender stereotypes they felt aligned with. In order to meet criteria, participants also described being asked how they hated themselves, their body, or to say that they had a “mental illness,” and according to participants, this is definitely not always the case:

Was like really negative for me *(crying)*, just because it was really felt like they were asking me to prove that I was trans or whatever. It was like a person who wasn’t trans who I don’t think really understood about that. I felt like I had to prove that I had a disorder ‘cause that's what the process is.

(Participant #2119, bisexual, trans/nonbinary, 25)

One participant described how they spent many years coming to terms with their identity, and being ok with themselves, and indicated how the medically prescribed process of transitioning sought to “undo” that progress, by attempting to make them admit they had a mental illness or that there was something wrong with who they were:

Another thing is for a medical professional to assume that you’re dysphoric. I mean, nothing- it makes you feel pretty awful about yourself to be seeing them when you’re not uncomfortable with your body and then sort of have it pushed on you that like- all of- it felt like you should- you should be or something…you know? I just felt like I wasn’t being listened to, even though I was very clearly articulating that this isn’t an issue for me…

(Participant #2819, gay transman, 32)

### Lack of Knowledge

All transgender participants described refusals of care by health care providers. Rationales included a “lack of knowledge” as a reason for declining to prescribe/treat due to uncertainty how hormones might “interact/affect” care, regardless of the reason for the visit, were also described:

I get a lot of “I’m not specialized. I never had training. I don’t know what to do…” and I understand that to a certain degree, but they’re also unwilling to go get the training and *there is a clinic that* literally has a website section dedicated to showing providers how incredibly easy it is to prescribe

(Participant #2819 gay transman, 32)

### Systems Level Barriers “The Message Is You Don’t Actually Exist”

System-level barriers are those related to how demographic information is collected. Multiple participants described how heteronormative assumptions in the collection and sharing of patient information disregarded their identities, potentially impacting care. Most participants reported personal experiences of having providers make improper assumptions about their sexual orientation or gender identity. The lack of adequate choices or options on forms created frustration for some, while others indicated the deeper impact regarding the stigmatization from a system that fails to recognize identities, or fails to provide opportunities to be appropriately addressed:

If a form doesn’t have what you identify as, asks you to identify but it doesn’t provide your identity anywhere and you have to write it in yourself**.** The message is, you don’t actually exist. Or, we don’t think you actually exist. Because if they did, that would be an option on the form. If it's not in the form, ok then you’re, you’re saying the message that you don’t think it's real.

(Participant #1004, gynasexual nonbinary, 49)

I haven’t changed my gender marker because as a non-binary person, I can’t change my gender marker on any of my ID to a congruent marker because currently here we only have two binary gender markers that do not match what my reality is. There's just no adequate system for communicating your gender and how you should be addressed…

(Participant #0719 queer nonbinary trans, 31)

Lack of communication among health care team members was another concern. Even when individuals had the opportunity to self-identify pronouns/gender/sexuality, often this failed to be documented and communicated:

They have never been in the years that I have been going there consistently gotten my name correct, even after it was legally changed, they were still calling me by the wrong name…… in front of the rest of the waiting room

(Participant #0719, queer nonbinary trans, 31)

### Access and Acceptance: Issues With Rural Life

Living rurally creates a number of unique issues for LGBTQ people. The lack of anonymity and confidentiality that comes along with living in a community where everyone knows each other was an expressed concern. Another issue described was when attitudes, norms, and beliefs of the community do not align with those of the patient; for example, living in a predominantly faith-based community, which is vocally anti-LGBTQ. One participant described how attitudes and behaviors in the community created uneasiness and lack of trust in the clinical setting, since the persons practicing discrimination in the larger community and were also serving as local health care providers:

I guess one of the big things that actually been a bit of a problem, problem in terms made me a bit hard at the time. The local clinic, I mean it staffed by largely local people, a small community. So I know a lot of them. And I’m somewhat out, well I’m out in certain places in our community, as being gender queer, in terms of my gender but in the clinic itself, no … .The issues through my work, I’ve had parents pull their students out of public schooling entirely rather than have them in my classroom.

(Participant #1004 gynasexual nonbinary, 49)

The increased social and geographic isolation of rural areas was also a concern. Fewer resources and supportive services exist overall, with even fewer LGBTQ-specific resources, supports, and services:

And just sometimes, even lack of services too, right? It's super tough trying to access a counsellor. The only counsellor for miles around is the faith-based counsellor that, probably don’t wanna discuss all of this, you know?

(Participant #0819, pansexual queer woman, 29)

Inadequate LGBTQ fertility and reproductive care, and supports, were also a concern. One participant described their experience as traumatizing and grossly inadequate:

When I was pregnant. That was a horrifying experience. Being pregnant as a transgender person was so traumatizing that I have- I have medical PTSD from my experience.

(Participant #050719 lesbian transwoman, 36)

### Giving Voice Back: Recommendations From the Community

Participants were asked what changes they would like to see in health care service delivery. The first priority identified was creating safe settings, free from assumptions, judgments, and discrimination. Participants all described critical holistic care aspects, including the importance of recognizing and understanding the intersection of identities and lived experiences. Improving the knowledge of the health care team, and eliminating heteronormative assumptions was also of primary importance, with all participants recommending ongoing training and education for all types of health service providers (nurses, doctors, mental health, etc.):

The biggest challenge is the healthcare system is woefully unprepared to take appropriate care of LGBTQ people. Like, the dream is healthcare providers to understand how our sexuality, or gender, our race, our class, our economic level, all are part of our health, you know? How they all intersect.

(Participant #1419 lesbian genderqueer, 38)

Participants also described the importance of building queer-friendly health and community services, including more supportive and inclusive, safe spaces in their community, where they could be “themselves.” Welcoming and inclusive community supports were identified as vital to the mental health and social well-being of persons who feel otherwise isolated and marginalized in their communities:

Places where trans people can be supported or whatever. So that if you can’t infiltrate schools and other communities very much like at least that there's another option in a holistic health sense, I guess. ‘Cause it's like one thing to just go to like a monthly support group and like your doctor appointment for like testosterone or whatever. But like if I have nowhere else I can be myself, then that's like- it would be bad for my health, I guess. So like having with like really regular community center-type thing would be amazing like the people can go regularly.

(Participant #2119 bisexual trans/nonbinary, 25)

Participants emphasized the importance of systemic changes and acknowledgment of identities, stating “They do exist!”

The participants also called for eradication of the persistent erroneous belief in health care regarding the presence of only two sexes. Health care settings adhering to such binary assumptions were described as silencing or pathologizing individuals identifying outside of those binaries, creating health care structures that maintain inequities. The need for health care providers to ensure their practices and colleagues support the dignity and worth of all people were also identified. Participants also reinforced the importance of professionals advocating against laws based on erroneous scientific notions, or that have clear ramifications for the health of populations. As one participant stated:

Like we need to openly acknowledge that the quote-unquote difference between sex and gender aka body versus brain is outdated, not backed by sociological understanding, not backed by medical understanding, that it is erasure, that it is dangerous, that it doesn’t accurately reflect the lived experiences of a large and invisible and marginalized portion of our population -and again, this is part of the holistic change. It needs to be acknowledged in every way possible from forms to medical care to medical education that- that these things are not as simple and binary as we treat them to be.

(Participant #0719 queer nonbinary trans, 31)

## Discussion

The purpose of this study was to gain a better understanding of the lived experiences of LGBTQ individuals within the health care system in Manitoba, and to highlight their visions for future care reformation. Through our analysis, we distilled the findings into six central themes related to the nature of experiences when accessing health care services, and how inadequate access, or lack of culturally appropriate care impacts overall health and well-being. These data contribute to the literature and knowledge on LGBTQ health, particularly within a Canadian and rural context. Importantly, we gained critical insights into the types of services needed for the education of current and future care providers, and for providing LGBTQ-friendly community services that address the broader determinants of health. Interventions that create opportunities to improve health care provider's understanding of the lived experiences of LGBTQ individuals are crucial in reducing stigma. This also includes recognition of how past experiences of discrimination, stigma, rejection, and risks of violence intersect, leading to avoidance of care and services and overall poorer health outcomes (NASEM, 2020). All participants described the negative impact of heteronormative assumptions made by health care providers for their physical and mental health. These findings are echoed in the literature, with many LGBTQ people continuing to have difficulty finding health care providers or settings where they feel safe, accepted, understood, and without fear of discrimination (Bell & Purkey, 2019; James et al., 2016; The National LGBT Health Education Centre, 2016). James et al.'s survey reported that when visiting a health care provider, one third of participants experienced negativity via verbal harassment and/or denial of treatment (2016). The continued pervasiveness of negative attitudes of health care providers toward LGBTQ patients has resulted in inadequate care and a deep mistrust of health care providers and the health care system, impacting access to health care and the overall health of the LGBTQ community (Lee & Kanji, 2017; NASEM, 2020).

Further to these negative experiences, the pathologization of identities by health care professionals in this study highlights an issue of a larger importance. Eckhert (2016) described the problematic nature of health care providers adopting pathologizing frameworks, and how these reinforce old biases where the patient's sexual orientation/gender becomes the problem, rather than considering how heteronormative biases in health care and the larger community have systematically created disparities for LGBTQ people. As Eckhert (2016) points out, the implications of this are two-fold: the harm that occurs at the patient level, with negative health care provider attitudes, resulting in poor patient care, and the larger issue of how this translates to a failure of the system addressing the health disparities of this population. The result is continued reinforcement of heteronormative biases in health care, creating challenges and damage for the LGBTQ community (Eckhert, 2016), as was evident in this study.

The lack of knowledge of health care providers remains a dominant issue. All participants in this study described how many health care providers lack the basic understanding of LGBTQ culture and terminology necessary to provide competent, sensitive care. Participants also described how heteronormative assumptions regarding gender or sexuality were the dominant discourse in most health care settings, rendering all other identities invisible. Such system-level barriers related to the collection of demographic information can result in identity erasure, disregarding of health risk factors, leading to an “invisibility” of LGBTQ identities. Many LGBTQ people as a result avoid seeking health care, reporting feelings of invisibility with their health care provider as a rationale (James et al., 2016; The National LGBT Health Education Centre, 2016). This invisibility also contributes to the heteronormative focus within the training, education, policy, and practices of the health care system overall (HCC, 2019; MAP, 2019).

Echoing previous research, our participants reinforced the need for upstream education of future care providers and the elimination of heteronormativity within the health care system (HCC, 2019; Schreiber et al., 2021). Undergraduate health professionals, including students of nursing, medicine, social work, and more, require foundational knowledge of LGBTQ persons, their health needs, and risk factors. Health care professionals require an understanding of how LGBTQ identities and lived experiences intersect, including how experiences with the health care system and care providers, potentially affect health (Schreiber et al., 2021; The National LGBT Health Education Centre, 2016). LGBTQ cultural competency education has been shown to increase the health care provider levels of comfort and knowledge, and patient perceptions of safety and satisfaction. Eliminating heteronormativity in preparatory programs facilitates improved knowledge, sensitivity, and awareness for new professionals entering practice (Porter & Krinsky, 2014).

Historically, most studies regarding LGBTQ health experiences were conducted with urban populations. Previous studies on rural LGBTQ experiences have identified issues that align with our findings, including experiences of stigma, social isolation, geographic distance, and the need for LGBTQ-specific services (MAP, 2019; Whitehead et al., 2016). The MAP (2019) reports delineated key issues regarding rural life for LGBTQ people in the United States. These findings aligned with the findings of this study, including how rural life serves to “amplify the impacts of both acceptance and rejection” (MAP, 2019, p. 1), meaning discrimination can have a more significant effect due to certain key commonalities of rural communities. Firstly, with smaller populations, differences become more noticeable, secondly, when communities are closely connected, rejection and acceptance in one area of life (eg, faith community) can impact other areas (eg, work) (MAP, 2019). As one participant identified, their work in the schools and knowing community members from that context affected their ability to access health care without fear of discrimination. Thirdly, when discriminated by a health care provider, the limited number of rural providers becomes even more limited. As another participant noted, traveling to another community was their only option after negative experiences in their own community. Finally, MAP noted how the social and geographic isolation results in few supportive resources and limited opportunities to create a supportive community that helps individuals withstand difficulties, including discrimination (2019). As participants noted, rural communities were lacking in LGBTQ allies, resources, and social connections specific to their needs. Given that social supports and access to health services are major health determinants, this is a major concern. Our respondents’ unanimous call for health care professionals to improve knowledge and sensitivity regarding LGBTQ populations takes on greater urgency in rural settings. Whitehead et al.'s (2016) study of rural LGBTQ health utilization also reinforced the need for greater rural provider education on creating safe environments and promoting LGBTQ inclusion. This was particularly important for transgender and nonbinary persons, as they often require significant travel for competent and sensitive care (Bell & Purkey, 2019). Other areas of education Whitehead et al., described as vital rurally also aligned with our findings, including increasing health care providers’ knowledge regarding known risk factors, lack of preventative health care in LGBTQ populations, as well as educates the LGBTQ population on the importance of disclosure, in order to overcome stigma (2016).

## Limitations

This study adds unique elements to a growing body of knowledge regarding the challenges for the LGBTQ community when accessing health care rurally, however, there are some limitations noted. One limitation of this study is the small sample size. Although the depth of data acquired in qualitative research necessitates smaller sample sizes, it also has potential to limit some transferability. However, given the specific and personal nature of this study, using a smaller sample size allowed the researchers to establish trust and fully engage with participants. Given the issues identified with health care trust, this was a critical step in obtaining accurate and meaningful data. Another major challenge of exploring health care experiences of LGBTQ populations remains the issue of only studying those who feel safe to self-identify, thus excluding a large closeted population (Lee & Kanji, 2017). Although this study highlights unique aspects regarding rural and remote communities, a rural focus might limit transferability to urban regions. However, the value of examining the challenges outside of urban, resource-rich contexts outweighs this limitation. This work draws attention to lesser-known issues faced by many LGBTQ persons residing outside of larger centers regarding the impact of attitudes and beliefs in less diverse settings, and the larger issue concerning the lack of rural resources overall. This is particularly relevant, as distances between communities and larger centers can be vast, creating significant geographical and financial barriers for many. Studies such as this can help to identify and address issues within these communities, creating opportunities for advocacy for funding and resources in deficient regions.

## Implications

Further research and education are needed to address the issues and challenges within this context. Education and cultural competency trainings for health care professionals are urgent priorities for rural service providers. Eliminating heteronormative assumptions, teaching person-centred approaches, and improving communication strategies in preparatory programs are crucial changes for addressing deficits (HCC, 2019). Health care professionals interact with individuals at their most vulnerable, thus education must consider the impact of intersectionality and recognize the unique experiences and challenges that rural LGBTQ minorities face (MAP, 2019; Whitehead et al., 2016). Re-evaluation of the inclusiveness of existing health care and community services, public communication, documentation, images presented, and language choices and terminology used remains critical (HCC, 2019). For maximum effectiveness, rather than duplicating urban programs and services, programs must consider the unique experiences, needs, and values of rural LGBTQ people (MAP, 2019).

## Conclusion

Stigma, discrimination, and a lack of knowledge remain pervasive issues for LGBTQ people when accessing health care services. Lack of appropriate rural resources and supports magnify these issues, negatively impacting physical and mental health, creating a further disadvantage. Continued advocacy for antidiscrimination laws regarding gender and sexuality remain a priority, as discrimination and refusals of service continue. Living rurally means LGBTQ people already have fewer health care options, have increased visibility, and may be geographically isolated from other community and social supports, resulting in avoidance of services and making self-advocacy difficult. Involving rural LGBTQ people in this research, gave us valuable insights into their unique rural health needs and experiences, and gave voice to the LGBTQ rural community.
